# Ultrasonography methods for predicting malignancy in canine mammary tumors

**DOI:** 10.1371/journal.pone.0178143

**Published:** 2017-05-22

**Authors:** Marcus Antonio Rossi Feliciano, Ricardo Andrés Ramirez Uscategui, Marjury Cristina Maronezi, Ana Paula Rodrigues Simões, Priscila Silva, Beatriz Gasser, Leticia Pavan, Cibele Figueira Carvalho, Júlio Carlos Canola, Wilter Ricardo Russiano Vicente

**Affiliations:** 1 Department of Animal Reproduction, UNESP—Universidade Estadual Paulista “Julio de Mesquita Filho”, School of Agrarian Sciences and Veterinary Medicine, Campus Jaboticabal, Jaboticabal, São Paulo, Brazil; 2 Department of Veterinary Clinics and Surgery, UNESP—Universidade Estadual Paulista “Julio de Mesquita Filho”, School of Agrarian Sciences and Veterinary Medicine, Campus Jaboticabal, Jaboticabal, São Paulo, Brazil; Colorado State University, UNITED STATES

## Abstract

The aim of this study was to evaluate and compare the efficacy of B-mode, Doppler, contrast-enhanced ultrasonography (CEUS), and Acoustic Radiation Force Impulse (ARFI) elastography in predicting malignancy in canine mammary masses. This was a prospective cohort study from 2014 to 2016, which included 153 bitches with one or more mammary masses. A total of 300 masses were evaluated by ultrasonography (B-mode, Doppler, CEUS, and ARFI) and subsequently classified as benign or malignant by histopathology. Each ultrasound parameters studied were compared between benign and malignant masses by Chi-square or Student tests and differences were considered significant when P < 0.01. For the variables that proved significant differences were estimated the cut-off point, sensitivity, specificity, accuracy, and area under curve (AUC) by receiver-operating characteristic curve (ROC) analysis in a logistic regression model using histopathological classification as reference, to assess and compare diagnostic performance of each technique. Out of 300 mammary masses evaluated 246 were classified as malignant and 54 as benign. B-mode measurements showed sensitivity 67.9%, and specificity 67.6% as malignancy predictors on canine mammary masses; Doppler indexes systolic (>21.2 m/s) and diastolic velocity (>4.8 m/s) sensitivity 79.2% and specificity 70.8%; CEUS wash-out time (<80.5 s) sensitivity 80.2% and specificity 16.7%; and ARFI elastography shear velocity (SWV > 2.57 m/s) sensitivity 94.7% and specificity 97.2% In conclusion B-mode and Doppler ultrasound evaluations may assist in malignancy prediction of canine mammary masses with moderate sensitivity and specificity, already the SWV was an great accurate predictor. Therefore, ARFI elastography exam inclusion in veterinary clinic oncology and research is highly recommended, since it allows fast, non-invasive, and complication-free malignancy prediction of canine mammary masses.

## Introduction

Mammary tumors are one of the most important disorders in women and bitches, with high morbidity and mortality, and similar biological behavior in both species [1,2]. Ultrasonography has become an important tool in neoplasm evaluation and, when combined with mammography, can aid in the diagnosis, differentiation, and prediction of malignancy in mammary tumors in human medicine [3,4,5].

Several reports have demonstrated the applicability and limitations of B-mode ultrasonography [6,7,8,9,10,11,12], Doppler [8,10,13,14,15], elastography [3,4,16,17,18,19], and contrast-enhanced ultrasonography (CEUS) [20,21,22,23] in the evaluation of breast cancer in humans and canines. However, no report has yet compared the efficacy of these ultrasonography techniques in predicting malignancy of mammary tumors.

It has been suggested that B-mode, Doppler, contrast-enhanced ultrasound, and elastography can aid in the diagnosis of malignancy in breast tumors, non-invasive diagnostics techniques of easy and fast execution, enabling immediate results to the medical and veterinary; nevertheless, these techniques are believed to have different diagnostic efficacy. Thus, the aim of this study was to evaluate and compare the efficacy of B-mode, Doppler, contrast-enhanced ultrasonography, and Acoustic Radiation Force Impulse (ARFI) elastography in predicting malignancy in canine mammary tumors.

## Materials and methods

This study was approved by the Ethics Committee in the Use of Animals of the School of Agrarian Sciences and Veterinary Medicine, UNESP–Universidade Estadual Paulista, Jaboticabal-SP, Brazil (protocol No 023705/12).

### Experimental design

A prospective cohort study developed between 2014 and 2016 included 153 bitches with one or more mammary masses that were brought by pet owners for care to the “Governador Laudo Natel” Veterinary Hospital, UNESP-Univ Estadual Paulista, Jaboticabal-SP, Brazil. Pet owners signed a consent form for their animal inclusion in this experiment. All animals and a total of 300 masses were evaluated by ultrasonography and subsequently classified as benign or malignant by histopathology [24,25].

### Ultrasonography exam

Ultrasonography (US) was performed by a single experienced veterinary sonographer prior to mastectomy and histological identification of tumor type, using a 9.0 MHz linear transducer and ACUSON S2000® equipment (Siemens, Munich, Germany). Each mammary mass was evaluated using the different ultrasonographic methods (B-mode, Doppler, ARFI elastography, and CEUS) in the order described below.

### B-mode ultrasonography

The mammary masses were evaluated by conventional ultrasonography according to: echotexture (homogenous or heterogeneous), echogenicity in relation to the adjacent and normal mammary tissue (hypo, hyperechoic, or mixed with solid or liquid components), contours/margins (defined or undefined), invasiveness (present or absent), and other findings (presence of cystic, anechoic, and hyperechoic areas or acoustic shadowing). Additionally, the length (cm), width (cm), and width/length ratio in longitudinal section and height (cm), width (cm), and width /height ratio in transverse sections were obtained.

### Doppler ultrasonography

Doppler color flow imaging enabled the visualization (present or absent) and localization (peripheral, central, or diffuse) of tumoral vascularization and the identification of the type of vessel (Perinodular—vessels around mass parenchyma, mosaic—random vascular points into the parenchyma, or network) present in the tumors.

In tumoral vascularization analysis by spectral Doppler, the angle between the Doppler beam and the vessel’s long axis did not exceed 60^o^. Color gain was adjusted to reduce excessive color noise when blood flow was too slow. A 2–4 mm gate (depending on the diameter of the vessel) with apertures was positioned at the center of the vessel to measure the flow’s spectral trace, spectral curve, and vascular indexes; which were obtained automatically following software identification of the ultrasonic scanner for each waveform. A minimum of three subsequent waves was used in the evaluation. The parameters studied were: systolic velocity (SV, cm/s), diastolic velocity (DV, cm/s), resistive index (RI = (Vmax–Vmin) ⁄ Vmax), characteristic (arterial or turbulent), and pattern (high, intermediate, or low resistivity) of blood flow [10].

### Contrast-enhanced ultrasonography (CEUS)

CEUS was performed using contrast-specific software (CADENCE®, Siemens, Munich, Germany) with secondary harmonic imaging and inverted pulse technique. After delineation of the mass area, the probe was held steadily and the adjustable parameters such as depth, gain, mechanical index (0.07–1.1; interval constant between different tumors), and focal zones were optimized and maintained. The contrast agent (SonoVue®, Bracco, Milan, Italy) was immediately administered as an intravenous bolus (0.1 mL, followed by 5 mL saline flush) via a catheter in the cephalic vein. Video clips were obtained for five minutes following bolus injection of contrast and recorded in the internal storage system for each mass assessed.

Microbubble perfusion and the dynamic enhancement of the image of each lesion were subsequently analyzed based on the presence or absence of contrast in the tumoral mass; perfusion time through wash-in time (WI seconds), time to enhancement peak (TP s), and wash-out time (WO s); and enhancement characteristics: 1) enhancement level relative to surrounding normal mammary tissue (hyper, iso, or hypo enhancement), 2) pattern (centripetal, centrifugal, or diffuse), 3) localization (central, peripheral, or diffuse), 4) internal homogeneity (homogeneous or heterogeneous), and 5) perfusion type (discreet, moderate, or increased) [21].

### ARFI elastography

Qualitative and quantitative analysis were performed using the VTIQ method of ARFI (virtual touch tissue imaging quantification, 2D-SWE technique) [3,19]. Qualitative ARFI resulted in greyscale images (elastogram) that were evaluated according to deformability (deformable or not deformable), whitish tones (bluish areas—less rigid) corresponded to more elastic tissues (soft) and darker tones (reddened areas, rigid not deformable tissues) to more rigid tissues (hard). Additionally, the quality of the examination was evaluated using the display device: homogeneous and greenish images to indicate high quality of the technique; and yellowish and heterogeneous images to indicate low quality of the technique. Quantitative evaluation consisted of a software function that determined shear wave velocity once the calliper was positioned on the mass parenchyma. Six measurements of different areas in each tissue randomly selected were used to determine the mass mean shear wave velocity (SWV m/s).

### Histopathological classification

Following ultrasonography evaluation, the animals were referred to the Department of Veterinary Clinics and Surgery for mastectomy. Samples of the mammary masses were collected for histopathology analysis and their macroscopic appearance evaluated. Multiple tissue fragments were fixed in 10% phosphate buffer formaldehyde solution (pH 7.4) and routinely processed for histopathology analysis prior to paraffin embedding. Tissue sections (5 μm) were mounted onto glass slides and stained with Haematoxylin and Eosin (HE).

The neoplasms were analyzed by single and experience pathologist under light microscopy and histologically classified as benign or malignant according to the criteria recommended by the World Health Organization [25]. Posterior classification and staging were made in accordance with the Consensus for diagnosis, prognosis, and treatment of Canine Mammary Tumors [24].

### Statistical analysis

Statistical analysis was performed using the software R, version 3.3.0 (R® foundation for statistical computing, Austria). Qualitative ultrasound variables were compared between benign and malignant masses by Chi-square test, quantitative variables by Student test and differences were considered significant when P-value < 0.01. For ultrasonography parameters that showed significance, the cut-off point, sensitivity, specificity, accuracy, and area under curve (AUC) were calculated using histopathological classification as a reference for receiver-operating characteristic curve (ROC) analysis in a logistic regression model aimed at assessing and comparing the diagnostic performance of each technique.

## Results

**Fig 1:**
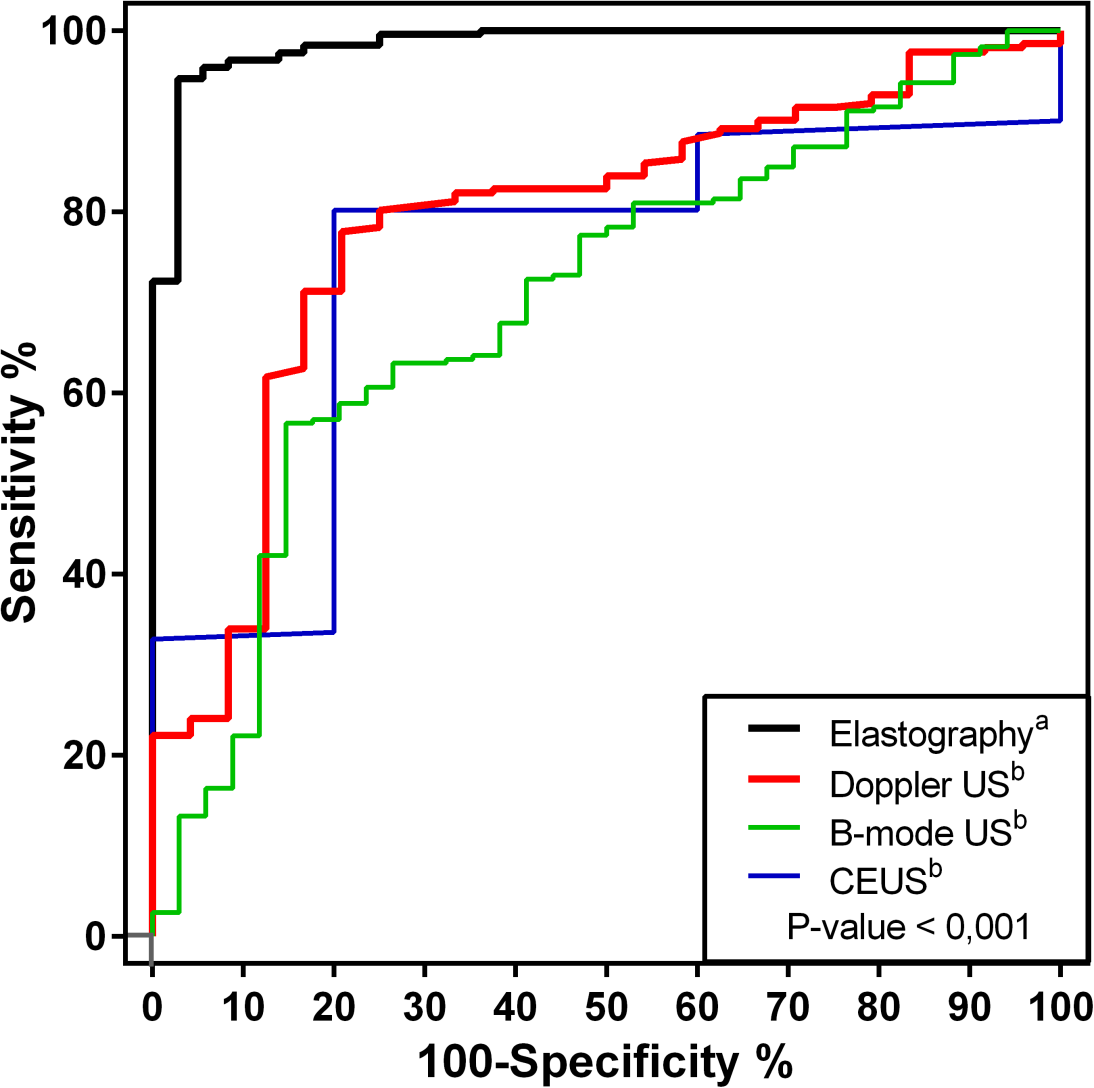
Receiver-operating characteristic curve comparing predictive sensitivity (%) and specificity (100-%) of different ultrasonography methods in determining malignancy in canine mammary masses using histopathological classification as a reference. Different letters indicate significant difference.

Out of the 300 mammary masses evaluated, 246 (82%) were histopathologically classified as malignant and 54 (18%) as benign and histopathological classification is detailed in Table 1. Ultrasonographic evaluation was performed without difficulties, intercurrences, or side effects. US findings are summarized in Tables 2 and 3, diagnostic performance variables in Table 4, and comparative receiver-operating characteristic curves in Fig 1.

**Table 1:** Histopathological classification of canine mammary tumors [24,25].

Classification	Type	Diagnosis	Number
**Malignant**	Carcinomas	Carcinoma in a mixed tumor	129
		Ductal carcinoma *in situ*	3
		Lobular carcinoma *in situ*	11
		Papillary carcinoma	27
		Tubular carcinoma	30
		Solid carcinoma	18
		Complex carcinoma	7
	Special type carcinomas	Malignant adenomyoepithelioma	8
		Secretory carcinoma	2
		Micropapillary carcinoma	5
		Anaplastic carcinoma	1
		Squamous cell carcinoma	2
		Inflammatory carcinoma	3
**Total Malignant**			**246**
**Benign**	Epithelial hyperplasia	Ductal hyperplasia	4
		Lobular Hyperplasia	7
	Benign neoplasm	Adenoma	5
		Fibroadenoma	1
		Benign mixed tumor	37
**Total Benign**			**54**

**Table 2:** Rate of qualitative variables evaluated by different ultrasonography methods (B-mode, Doppler, contrast-enhanced ultrasonography and ARFI elastography) in malignant and benign canine mammary tumors.

Variables	Parameter	Benign	Malignant	P-value
**B-Mode ultrasonography**				
**Echotexture**	Homogenous (%)	31	50	1.0000
	Heterogeneous (%)	69	31	
**Echogenicity**	Hypoechoic (%)	50	30	0.0450
	Hyperechoic (%)	0	2	
	Mixed (%)	50	68	
**Contours or margins**	Defined (%)	97	99	0.4210
	Undefined (%)	3	1	
**Invasiveness**	Present (%)	0	0	1.0000
	Absent (%)	100	100	
**Doppler ultrasonography**				
**Vascularization**	Present (%)	86	67	0.0065*
	Absent (%)	14	33	
**Localization**	Peripheral (%)	52	75	0.1210
	Central (%)	8	4	
	Diffuse (%)	40	21	
**Vessel type**	Perinodular (%)	31	17	0.0318
	Mosaic (%)	31	58	
	Network (%)	38	25	
**Characteristics**	Arterial (%)	95	100	0.0535
	Turbulent (%)	5	0	
**Patterns**	High resistivity (%)	50	25	<0.0001*
	Intermediate (%)	14	63	
	Low (%)	36	12	
**Contrast-enhanced ultrasonography**				
**Enhancement level**	Hyperenhancement (%)	15	0	0.1083
	Hypoenhancement (%)	34	80	
	Isoenhancement (%)	51	20	
**Pattern**	Centripetal (%)	10	20	0.2149
	Centrifugal (%)	34	60	
	Diffuse (%)	56	20	
**Localization**	Central (%)	5	0	0.7242
	Peripheral (%)	54	40	
	Diffuse (%)	41	60	
**Homogeneity**	Homogeneous (%)	2	0	1.0000
	Heterogeneous (%)	98	100	
**Perfusion type**	Discreet (%)	15	0	0.1083
	Moderate (%)	34	80	
	Increased (%)	51	20	
**ARFI Elastography**				
**Deformability**	Deformable (%)	24	66	<0.0001*
	Not Deformable (%)	76	34	

*Difference considered significant (Chi-square test).

**Table 3:** Mean ± SD of quantitative variables evaluated by different ultrasonography methods (B-mode, Doppler, contrast-enhanced ultrasonography and ARFI elastography) in malignant and benign canine mammary tumors.

Variables	Parameter	Benign	Malignant	P-value
**B-Mode ultrasonography**				
**Measures**	Longitudinal width (cm)	0.78 ± 0.90	1.18 ± 1.07	0.0006*
	Longitudinal length (cm)	1.64 ± 1.26	2.48 ± 1.63	0.0002*
	Width/length ratio	0.48 ± 0.37	0.45 ± 0.15	0.3681
	Transverse height (cm)	1.54 ± 1.15	1.97 ± 1.54	0.1595
	Transverse width (cm)	0.72 ± 0.76	1.43 ± 1.23	0.0001*
	Width /height ratio	0.48 ± 0.28	0.97 ± 0.93	0.0004*
**Doppler ultrasonography**				
**Vascular indexes**	Systolic velocity (cm/s)	18 ± 11	37 ± 27	<0.0001*
	Diastolic velocity (cm/s)	5.1 ± 3.1	8.8 ± 8.7	0.0099*
	Resistive index	0.71 ± 0.1	0.76 ± 0.1	0.0240
**Contrast-enhanced ultrasonography**				
**Perfusion times**	Wash-in time (s)	13 ± 7.6	9.1 ± 5	0.1583
	Wash-out time (s)	20 ± 7.2	15 ± 5.7	0.0650
	Time to peak (s)	81 ± 17	64 ± 22	0.0819
**ARFI Elastography**				
**Shear wave velocity**	SWV (m/s)	1.5 ± 0.73	5.8 ± 2.4	<0.0001*

cm: centimeters; s: seconds; m: meters; SD: standard deviation

*difference considered significant (Student test).

**Table 4:** Predictive performance variables (%) of different ultrasonography methods in determining malignancy in canine mammary tumors using histopathological classification as a reference.

Parameters	Cut-off	Sensitivity	Specificity	Accuracy	AUC
**B-Mode ultrasonography**					
**Longitudinal width (cm)**	>1.28	70.76	61.76	69.63	68.10
**Longitudinal length (cm)**	>0.52	71.19	61.76	70.00	69.3
**Transverse width (cm)**	>0.66	67.84	73.53	68.58	72.10
**Width/height ratio**	>0.49	61.95	73.53	61.92	68.50
**Doppler ultrasonography**					
**Systolic velocity (cm/s)**	>21.2	77.83	79.17	77.54	78.70
**Diastolic velocity (cm/s)**	>4.8	67.92	62.50	65.68	67.30
**Presence of vascularization**	N/A	86.18	33.33	79.43	N/A
**Intermediate resistivity**	N/A	85.85	62.50	83.47	N/A
Contrast-enhanced ultrasonography*					
**Wash-out time (s)**	<80.5	80.15	16.67	77.37	74.00
**ARFI Elastography**					
**Shear wave velocity (m/s)**	>2.57	94.72	97.22	95.04	98.50
**Deformable tissues**	N/A	75.61	66.67	74.47	N/A

AUC: area under the curve; N/A: data not available; cm: centimeters; s: seconds; m: meters

*even without significant difference, the wash-out time of contrast-enhanced ultrasonography was analyzed for compared US techniques.

### B-mode ultrasonography

The B-mode US variables echotexture, contours/margins, invasiveness, echogenicity, findings, mass width/length ratio in longitudinal section and thickness in transverse section were not significantly (P>0.01) correlated to malignancy. Already, mass length and width in longitudinal section, and width and width/height ratio in transverse section were significantly (P<0.001) greater in malignant tumors and these B-mode variables showed a mean sensitivity 67.9%, specificity 67.6%, accuracy 67.5% and AUC 69.5% as malignancy predictors on canine mammary masses (Tables 1–3).

### Doppler ultrasonography

**Fig 2:**
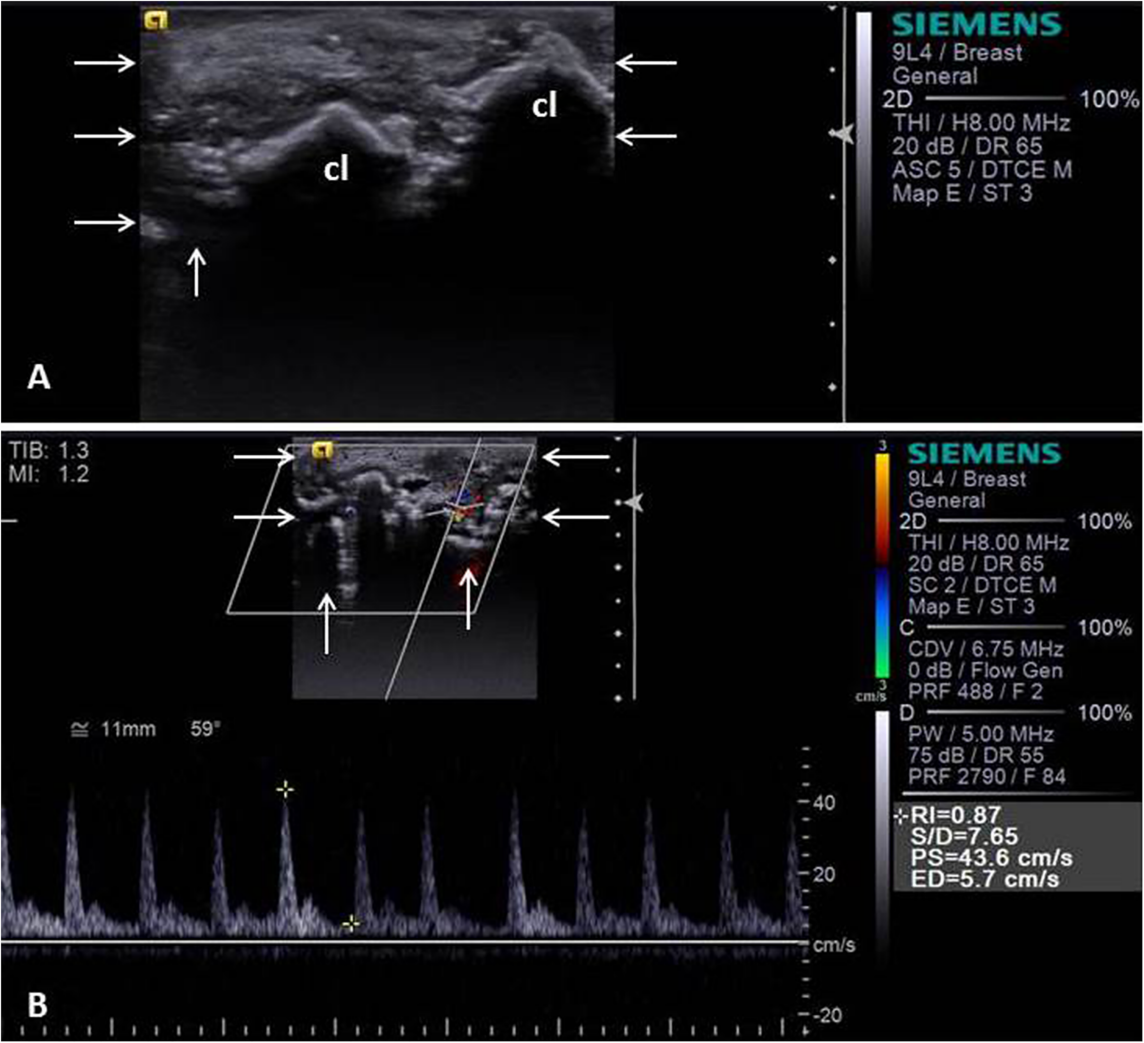
B-mode and Doppler ultrasonographic image of a canine mammary tumor—Solid carcinoma in female dog. B-mode image of mammary tumor (arrows) with presence of intratumoral calcification (cl) (A). Spectral Doppler ultrasonographic image of mass (arrows) with tumoral neovascularization and waveforms highlighting blood flow with indicated values of malignancy (high resistance and values on the right hand side) (B).

Color flow Doppler imaging revealed that malignant tumors showed higher proportion (P<0.01) of vascularization and intermediate resistivity pattern. This technique resulted on a mean sensitivity 86.0%, specificity 47.9% and accuracy 81.5% as malignancy predictors on canine mammary masses. In turn, Doppler spectral vascular indexes SV and DV were greater (P<0.01) in malignant tumors and showed a mean sensitivity 79.2%, specificity 70.8%, accuracy 71.6% and AUC 73.0% as malignancy predictors on canine mammary masses. Another blood flow characteristics and RI did not show significant (P>0.01) correlation with malignancy (Tables 1–3, Fig 2).

### Contrast-enhanced ultrasonography

**Fig 3:**
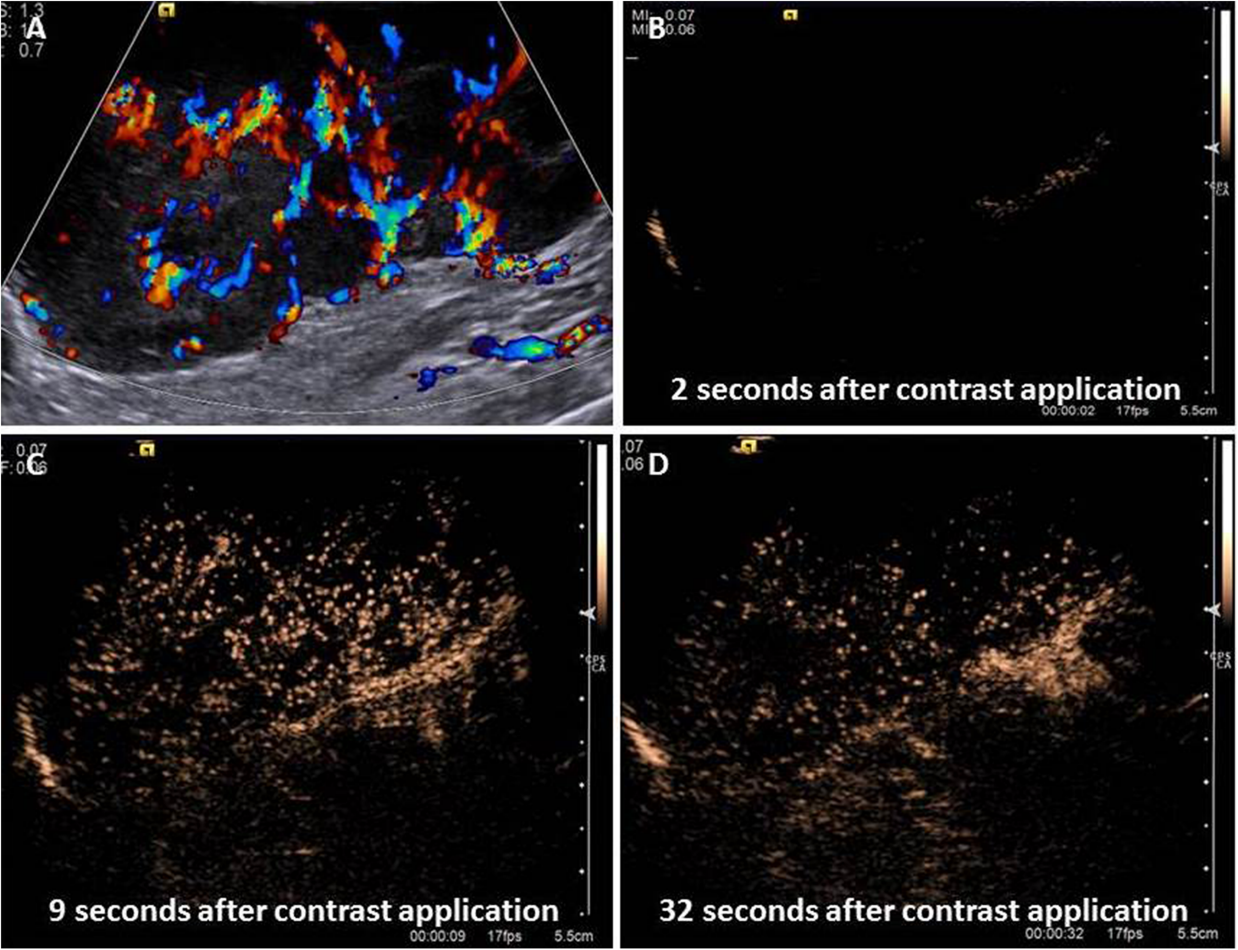
Contrast-enhanced ultrasonography (CEUS) image of a canine mammary tumor—Solid carcinoma in female dog. Color Doppler in mammary neoplasm (A) highlighting the presence of neovascularization in the tumor. The contrasted technique was applied to evaluate capillarization characteristics of the tumor: (B)—absence of contrast, (C) peak enhancement and diffuse enhancement, and (D) contrast wash-out. Time acquisition after contrast medium injection is located at the bottom of the right side of the images (B, C and D).

Contrast-enhanced ultrasonography enabled the evaluation of capillarization (macro and microcirculation) of the mammary tumors (Fig 3). However, none of the CEUS parameters evaluated showed significant (P>0.01) correlation to mammary mass malignancy. However, ROC analysis were applied for WO (P = 0.065) for comparative diagnostic performance study of US methods. WO lowers than 80.5 s showed sensitivity 80.2%, specificity 16.7%, accuracy 77.4% and AUC 74.0%.

### ARFI elastography

**Fig 4:**
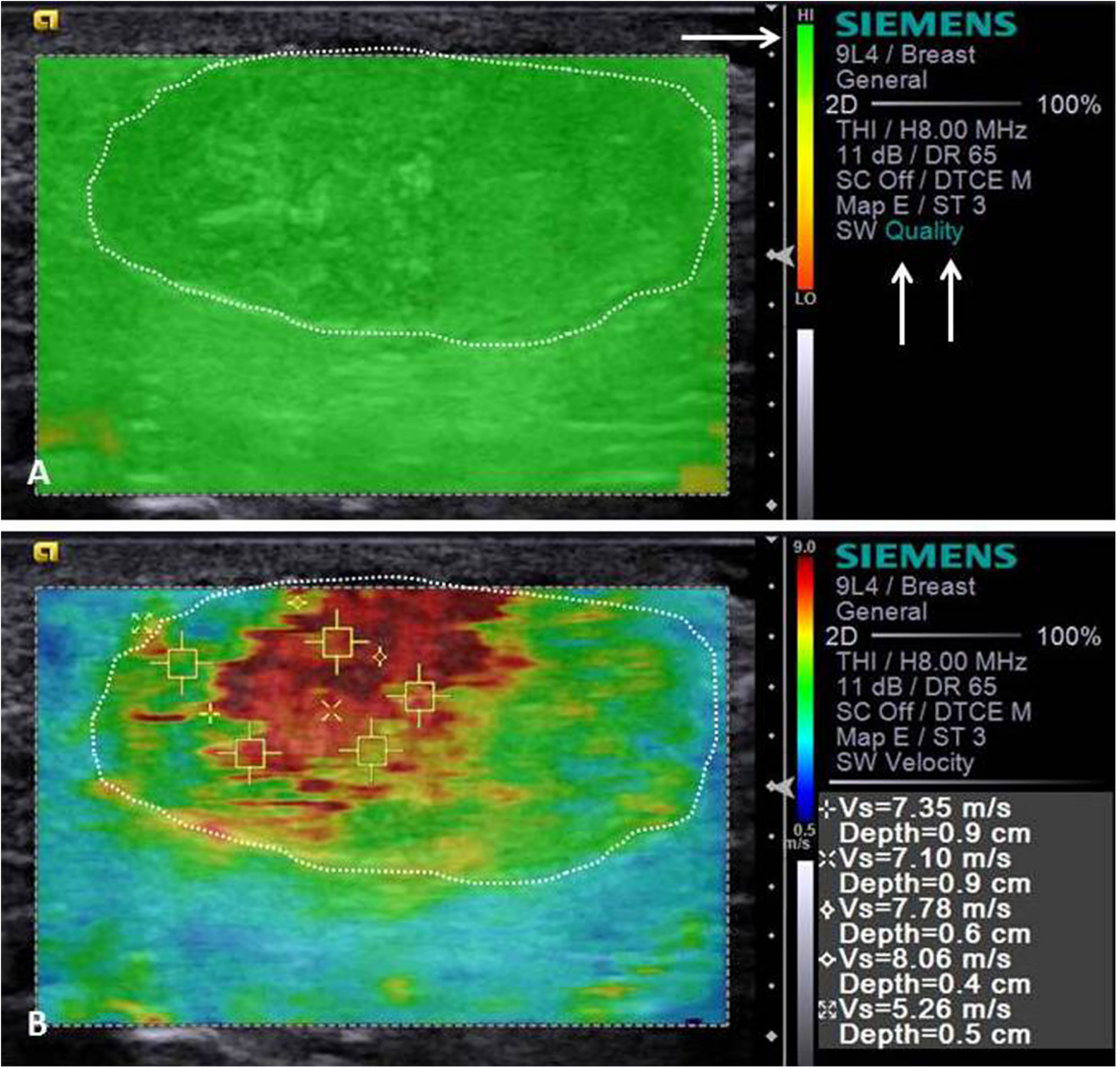
Acoustic radiation force impulse (ARFI) elastography image showing stiffness characteristics in a canine mammary tumor—Carcinoma in a mixed tumor in female dog. High quality image map (arrows) of VTIQ shows a homogeneous green picture of the lesion (white dotted line delimiting the neoplasm) (A). In VTIQ shear wave velocity mode (B), SWV values in the lesion (white dotted line delimiting the neoplasm) were measured and repeated five times. In elastogram (VTIQ qualitative—B), the image of the neoplasm (arrows) is heterogenous and not deformable, with rigid tissue (reddened areas in the central region) and with soft tissue (greenish areas in the peripheral region).

**Fig 5:**
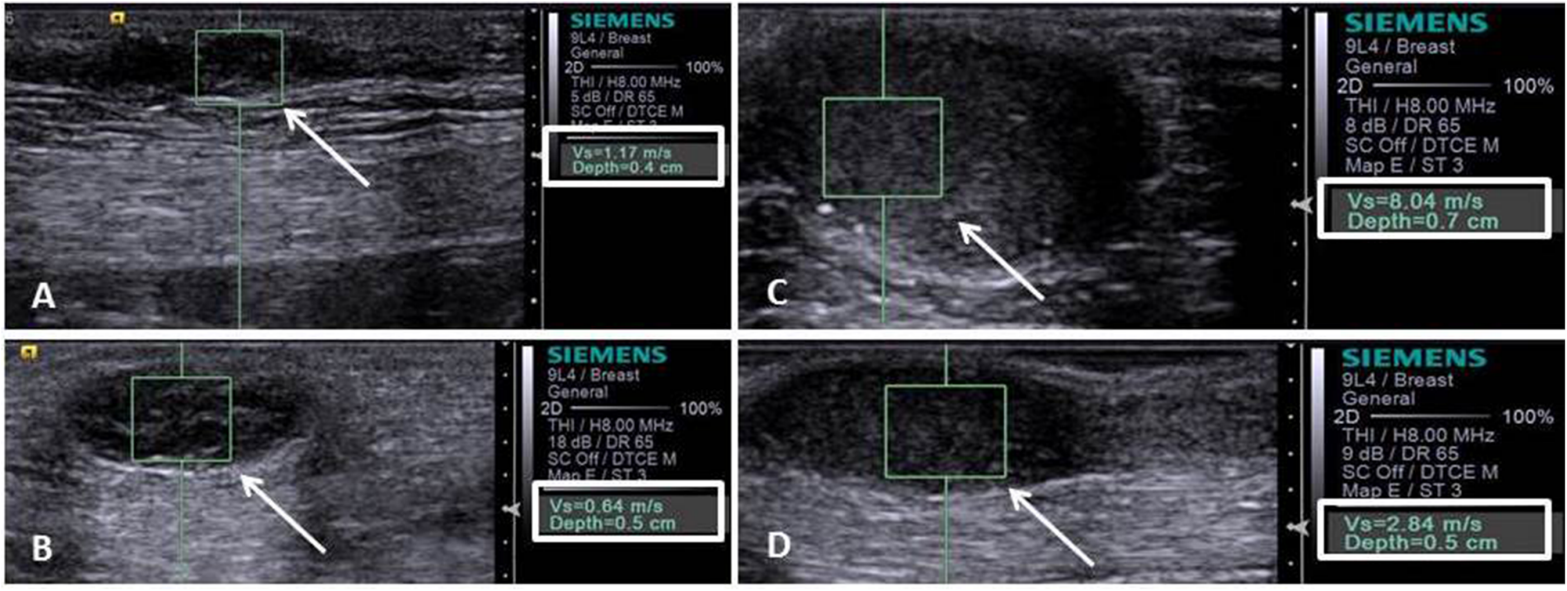
Image of the acoustic radiation force impulse (ARFI) elastography, which shows the values of shear velocity (right bottom corner) of images in the mammary tumors (arrows): A)–mixed benign tumor—shear velocity of 1.17 m/s; B) mixed benign tumor—shear velocity of 0.64 m/s; C) carcinoma in a mixed tumor (grade III)—shear velocity of 8.04 m/s; and D) carcinoma in a mixed tumor (grade II)—shear velocity of 2.84 m/s.

Tissue deformability was found to be proportionally higher (P<0.01) in malignant masses. Mostly red (dark) masses on the elastogram image (not deformable) were indicative of malignancy with sensitivity 75.6%, specificity 66.7%, and accuracy 74.5%. In turn, quantitative elastography enabled the mammary masses SWV determination, which was significantly (P<0.01) higher in malignant tumors. The ROC analysis indicated that an SWV > 2.57 m/s shown to be the best (P <0.01) malignancy predictive tool of canine mammary masses (Fig 1), with sensitivity 94.7%, specificity 97.2%, accuracy 95.0% and AUC 98.5% (Tables 1–3, Figs 4 and 5).

## Discussion

Ultrasonography evaluation of malignancy in mammary tumors in bitches showed variable efficacy in relation to standard methods of malignancy diagnosis. B-mode variables and vascular indexes evaluated by Doppler enabled the prediction of malignancy with moderate sensitivity, specificity and accuracy, CEUS evaluation showed high sensitivity but low specificity, while stiffness evaluation by ARFI elastography resulted in an exceptionally effective technique for malignancy prediction in canine mammary masses.

Based on the results from this study, quantitative ARFI elastography proved to be the best method of ultrasonographic prediction of malignancy in mammary masses. VTIQ ARFI elastography enables the quantitative evaluation of tissues stiffness, resulting in shear wave velocity (waves that return from target tissues) estimation. This modern tool showed the highest diagnostic efficacy in differentiating malignant and benign mammary masses, corroborating with recent reports [3,4,18,19,26,27,28,29] and the only reliable parameter. Cut-off values above 2.57 m/s showed an impressive 95% diagnostic accuracy and 98% AUC, with adequate sensitivity and specificity values. The cut-off values in this study were lower than those from previous reports; however, with greater sensitivity and specificity for women. In human medicine, some authors [19,26,30,31,32] have reported cut-off values ranging from 2.9–6.4 m/s, which have been associated to 76–91% sensitivity and 80–95% specificity, close values to the observed in the present study and which corroborate the effectiveness of the ARFI technique applied in evaluation of mammary tumors in canines.

The elastogram characteristics obtained in this study were adequate in the diagnosis of malignancy and similar to those previously described in benign mammary lesions in women [4,16,17,18] and bitches [3], with whitish tones (less rigid) in benign and darker tones (rigid not deformable tissues) in the malignant masses. The greater stiffness observed in malignant tumors is a consequence of the stromal reaction induced by the mammary carcinoma, which is associated with increased levels of collagen [3].

B-mode ultrasonography showed low efficacy in the differentiation of mammary tumors and findings such as invasiveness, irregular contours, acoustic shadowing, and echotexture were not indicative of malignancy; in disagreement with some reports that have considered these characteristics to be indicative of malignant tumors [6,7,8,9,11]. Corroborating the results from this study, B-mode ultrasonography has been considered to be a technique with low specificity (e.g. compared to mammography) when used as an isolated method of evaluation [14,33]. Additionally, this low specificity can be justified by clinical and biological profile [34,35] and histopathological variability of each tumoral type [10], ie, the morphological and structural heterogeneity of benign and malignant tumor in humans and canines, besides the presence of nonspecific characteristics theses masses, makes it difficult the differentiation of tumor types by the B-mode image.

Malignant tumors are often larger that benign ones due to parenchyma alterations (e.g. secondary tissue lesion such as edema, necrosis, calcification, and hemorrhage) that produce liquid and solid components at echogenicity evaluation [10,12,36,37]. These assertions corroborate with the larger size (longitudinal length and width, and transverse width and width/height ratio) observed in the malignant masses. Transverse width/height ratios in breast cancer (Japan Society of Ultrasonics in Medicine) [38] and cut-off values greater than 0.7 have been described [12] as malignancy predictor tools, with 56.3% sensitivity and 92.9% specificity, similarly to the results obtained in the present study.

Color and Spectral Doppler (vascular index and tracing characteristics) have been shown to be important ultrasonography techniques in the differentiation of malignant and benign tumors [10,13,15,39,40]. High values of SV and DV observed in the present study have been described as satisfactory indicators of malignancy in mammary masses in humans [13,39,40,41,42] and animals [10]. Furthermore, the findings from this study on high/low resistivity patterns are suggestive of malignancy, as previously described [8,14,15], and may be correlated to the presence of tortuous vascular networks in malignant tumors [14] and demonstrate the influence of neoplastic vascular organization (neovascularization characteristics) in tumoral nutrition [10,14]. It is important to note that diastolic velocity and blood flow patterns had not yet been described in the literature as predictors of malignancy in mammary tumors.

Contrast-enhanced ultrasonography proved ineffective in the differentiation of mammary tumors; however, it proved useful in the identification of tumoral macro and microcapillarization. These findings differ from most reports on CEUS diagnostic efficacy in the characterization of mammary masses in humans [20,21,22,23], which have suggested this technique to be an acceptable predictor of malignancy. These divergent results may be due to the limited samples of benign neoplasm in this study and/or the differences in imaging methods. However, despite the low diagnostic efficiency, these results provide novel values for CEUS in canine mammary masses. A high degree of contrast enhancement has been considered as an indicative of benignity [21] and was proportionally higher in the benign masses analyzed in the present study. This increase in intensity in benign masses has been correlated to inflammation and fibroadenomas, probably due to inadequate intratumoral angiogenesis and, consequently, insufficient capillary network to support tissue development [43].

## Conclusions

In conclusion B-mode and Doppler ultrasound evaluations may assist in malignancy prediction of canine mammary masses with moderate sensitivity and specificity, already the SWV was an great accurate predictor. Therefore, ARFI elastography exam inclusion in veterinary clinic oncology and research is highly recommended, since it allows fast, non-invasive, and complication-free malignancy prediction of canine mammary masses.
